# Pyrosequencing analysis for rapid and accurate detection of clarithromycin resistance-associated mutations in Iranian *Helicobacter pylori* isolates

**DOI:** 10.1186/s13104-023-06420-0

**Published:** 2023-07-06

**Authors:** Helia Alavifard, Ali Nabavi-Rad, Kaveh Baghaei, Amir Sadeghi, Abbas Yadegar, Mohammad Reza Zali

**Affiliations:** 1 Foodborne and Waterborne Diseases Research Center, Research Institute for Gastroenterology and Liver Diseases, Shahid Beheshti University of Medical Sciences, Tehran, Iran; 2 Basic and Molecular Epidemiology of Gastrointestinal Disorders Research Center, Research Institute for Gastroenterology and Liver Diseases, Shahid Beheshti University of Medical Sciences, Tehran, Iran; 3 Gastroenterology and Liver Diseases Research Center, Research Institute for Gastroenterology and Liver Diseases, Shahid Beheshti University of Medical Sciences, Tehran, Iran

**Keywords:** *Helicobacter pylori*, Antibiotic resistance, Clarithromycin, Gene mutation, Pyrosequencing, Sanger sequencing

## Abstract

**Background:**

Treatment of *Helicobacter pylori* (*H. pylori*) infection has become challenging following the development of primary antibiotic resistance. A primary therapeutic regimen for *H. pylori* eradication includes clarithromycin; however, the presence of point mutations within the 23S rRNA sequence of *H. pylori* contributes to clarithromycin resistance and eradication failure. Thus, we aimed to develop a rapid and precise method to determine clarithromycin resistance-related point mutations using the pyrosequencing method.

**Methods and results:**

*H. pylori* was isolated from 82 gastric biopsy samples and minimal inhibitory concentration (MIC) was evaluated using the agar dilution method. Clarithromycin resistance-associated point mutations were detected by Sanger sequencing, from which 11 isolates were chosen for pyrosequencing. Our results demonstrated a 43.9% (36/82) prevalence in resistance to clarithromycin. The A2143G mutation was detected in 8.3% (4/48) of *H. pylori* isolates followed by A2142G (6.2%), C2195T (4.1%), T2182C (4.1%), and C2288T (2%). Although the C2195T mutation was only detected by Sanger sequencing, the overall results from pyrosequencing and Sanger sequencing platforms were comparable.

**Conclusions:**

Pyrosequencing could be used as a rapid and practical platform in clinical laboratories to determine the susceptibility profile of *H. pylori* isolates. This might pave the way for efficient *H. pylori* eradication upon detection.

## Introduction

More than half of the world’s population is estimated to be infected with *Helicobacter pylori* (*H. pylori*) [1]. This microorganism is a Gram-negative, microaerophilic, spiral-shaped recalcitrant pathogen that can survive in the harsh and ever-changing environment of the human gastric mucosa [2]. Compared to *H. pylori*-infected patients, accumulating evidence suggests that *H. pylori* eradication dramatically decreases the risk and incidence of gastric cancer development worldwide [3, 4]. This has led to the recommendation of the test-and-treat strategy for dyspeptic patients, representing a cost-effective approach based on the treatment of all *H. pylori*-infected patients [5]. Therefore, the efficacy of various *H. pylori* eradication regimens has gained extreme attention for the prevention and treatment of gastric diseases [6].

Effective treatment of *H. pylori* infection has encountered major obstacles due to the high prevalence of single- and multi-drug resistant *H. pylori* strains [7]. Notably, the prevalence of antibiotic resistance in *H. pylori* has reached alarming levels worldwide, and several studies have demonstrated that primary resistance to clarithromycin is a major factor in therapeutic failure [4]. Clarithromycin resistance is mostly associated with point mutations in the 23S ribosomal RNA (rRNA) gene of *H. pylori* strains [8]. The A-to-G point mutations (A2142G and A2143G) within the V domain of the 23S rRNA gene have been demonstrated as the most frequent mutations; however, little is known about the clinical significance of other point mutations [9, 10]. Owing to the crucial importance of clarithromycin in eradicating *H. pylori* infection and the significance of preventing serious clinical consequences of clarithromycin resistance, rapid and accurate diagnostic methods to determine such macrolide resistance appear mandatory. Currently, several molecular assays have been introduced to determine the antibiotic resistance profile of *H. pylori* isolates. Sanger sequencing has been widely used to detect mutations conferring antibiotic resistance in *H. pylori* strains. However, this approach is time-consuming, labor-intensive, and expensive [11]. Recently, pyrosequencing, a rapid method of sequencing relatively short DNA targets, has been used in a number of microbiological applications [12]. This is a novel bio luminometric method of sequencing that has potential advantages over conventional sequencing methods when merely a short target sequence is required. Pyrosequencing assay can be considered a cost-effective, robust, sensitive, and high-throughput mutation detection assay [13]. In this study, we aimed to develop a pyrosequencing screening assay for the detection of clarithromycin resistance-associated mutations within the 23S rRNA gene of *H. pylori* isolates. We also compared the results of the pyrosequencing method with the results of Sanger sequencing.

## Materials and methods

### Collection of gastric biopsy samples

Biopsy specimens were taken from 82 patients with upper gastrointestinal symptoms during gastric endoscopy at the Taleghani Hospital in Tehran. Briefly, three antral biopsies were obtained from the stomach of each patient for *H. pylori* isolation, rapid urease test (RUT) and histopathological examination. Infection with *H. pylori* in these patients was primarily evaluated by histological observations and RUT, and confirmed by culture and PCR as previously described [14]. Our patient selection criteria excluded individuals taking any antibiotics, proton pump inhibitors, or H_2_ blocker within two weeks before endoscopy. The biopsies for culture were instantly placed in a transport medium consisting of thioglycolate, 1.3 g/L agar (Merck Co., Hamburg, Germany), and 3% yeast extract (Oxoid Ltd., Basingstoke, UK) and delivered to the Helicobacter Laboratory. All participants and/or their legal guardians signed written informed consent before enrollment in the study. The study protocol was approved by the Institutional Ethical Review Committee of the Research Institute for Gastroenterology and Liver Diseases at Shahid Beheshti University of Medical Sciences (Project No. IR.SBMU.RIGLD. REC.1395.878).

### *H. pylori* culture and identification

The gastric biopsy samples were homogenized using a tissue homogenizer (Kontes, Vineland, New Jersey) and cultured on Brucella agar plates containing 7% (v/v) horse blood, 10% fetal calf serum (FCS), Campylobacter-selective supplement (vancomycin 2.0 mg, polymyxin B 0.05 mg, trimethoprim 1.0 mg), and amphotericin B (2.5 mg/L). The cultured plates were incubated in a CO_2_ incubator for 3–7 days in a microaerophilic atmosphere at 37°C. The grown microorganisms were recognized as *H. pylori* by colony morphology, Gram staining, and positive results from urease, catalase, and oxidase tests. The genus- and species-specific PCR was displayed using specific primers for the *H. pylori* 16S rRNA and *ureC* (*glmM*) genes to confirm *H. pylori*’s presence in the bacterial culture [14].

### Sensitivity of *H. pylori* strains to clarithromycin

The agar dilution method was used to determine the minimum inhibitory concentrations (MIC) of clarithromycin. Mueller-Hinton agar medium supplemented with 7% (v/v) horse blood was used as the culture medium. Bacterial suspension equivalent to McFarland turbidity standard no. 3 was made ready and cultured on the plates. Agar dilution MIC tests were performed according to the standard method recommended by the Clinical and Laboratory Standards Institute (CLSI) [15]. The plates contained serial dilutions of clarithromycin with concentrations ranging from 0.06 to 64 mg/L. MICs and antibiotic susceptibility were recorded after 3 days of incubation in a microaerophilic atmosphere.

### Sanger sequencing of the 23S rRNA gene

The primers used for the Sanger sequencing of 23S rRNA gene are indicated in our previously published article [14]. The forward and reverse primers were used to amplify each fragment and the purified PCR products were sequenced with an automated sequencer (Macrogen, Seoul, Korea) according to the manufacturer’s instructions. The results were processed as we announced in our previously published article [14].

### Pyrosequencing primer design for 23S rRNA gene

Forward, reverse, and sequencing primers are required for pyrosequencing assay. To design the forward and reverse primers, we first performed sequence alignments of the 23S rRNA gene from various *H. pylori* strains and using the complete 23S rRNA sequence of *H. pylori* UA802 (U27270.1). The 1041 base pair (bp) region of the *H. pylori* genome, which comprises all of the previously published mutations, was divided into three parts and the primers were designed for each part separately using CLC Sequence Viewer 8 (Qiagen, Hilden, Germany) and Gene Runner Version 3.05 software (Hastings Software Inc., Hastings, NY, USA). A short 23S rRNA target region was chosen to allow for efficient rapid-cycle PCR. The PCR primers were labelled with biotin at the 5^’^ end. The sequencing primer was designed to be located just upstream of the mentioned mutation regions to analyze the clarithromycin resistance-related mutations. Briefly, primers for mutational analysis were designed by the Pyromark Q48 Advanced Software (Qiagen, Hilden, Germany). The primers for PCR amplifications and pyrosequencing are presented in Table 1.

**Table 1:** Oligonucleotide sequences used for *H. pylori* detection, and the forward, reverse and sequencing primers designed for pyrosequencing the respective amplicons of the 23S rRNA gene of *H. pylori* isolates

Method	Primers	Oligonucleotide sequence (5’- 3’)	References
PCR	16S rRNA	F: GGCTATGACGGGTATCCGGC R: GCCGTGCAGCACCTGTTTTC	[14]
	*glmM*	F: GGATAAGCTTTTAGGGGTGTTAGGGG R: GCTTACTTTCTAACACTAACGCGC	[14]
	23S rRNA (Part 1)	F: TGGGAGCTGTCTCAACCAGAGAT Bio-R: ACTTCAAAGCCTCCCACCTATCC	This study
	23S rRNA (Part 2)	F: TGCGCAGGATAGGTGGGA Bio-R: GACCGCCCCAGTCAAACTAC	This study
	23S rRNA (Part 3)	F: GTTTGGCACCTCGATGTCGG Bio-R: GATGCTCTTGGCAGACAACTGG	This study
Sequencing	Seq (2142/43)	ACCCGCGGCAAGACG	This study
	Seq (2182/2195)	TTACTACAACTTAGCACTGC	This study
	Seq (2288)	GATGTTTCTGTTAGCTAACT	This study

### PCR amplification

Extracted DNAs from subcultures of each *H. pylori* strain were PCR amplified using an Eppendorf thermocycler (Eppendorf, Hamburg, Germany) for each part in a volume of 25 µL containing 2.5 µl 1X PCR buffer, 1 pmol of primers, 2 µL of DNA template (approximately 200 ng), 100 mM of dNTPs, 2 mM of MgCl2, and 1.5 U/µL SuperTaqTM DNA polymerase (HT Biotechnology Ltd., Cambridge, UK). PCR amplification was performed in the following condition: one cycle at 94℃ for 4 min followed by 45 cycles at 94℃ for 1 min, 56℃ for 30 s, and 72℃ for 30 s. A final elongation step was at 74℃ for 10 min.

### Template preparation and pyrosequencing

Pyrosequencing was performed using PyroMark Q48 Advanced CpG Reagents (4 × 48) (Qiagen, Hilden, Germany) on a PyroMark Q48 autoprep instrument following the manufacturer’s instructions (Cat No./ID: 974,230). For each pyrosequencing run, 10 µL of biotinylated PCR product were bound to 3 µl PyroMark Q48 Magnetic Beads and were pipetted into the correct wells of the PyroMark Q48 Disc. 60 µL of sequencing primer were downloaded on the instrument in the mentioned cartridge. There are two other cartridges for nucleotides and other reagents such as denaturation solution, enzyme, substrate, and annealing buffer. All additional steps were carried out automatically by the instrument. Results were automatically analyzed using PyroMark Q48 Autoprep software.

## Results

### Diagnosis of *H. pylori* isolates

A total of 82 biopsy samples were obtained from *H. pylori*-infected patients in the age range of 25–75 years old (Table 2). Biopsy samples were taken from the gastric antrum of participating patients. Thirty patients were male (63.5%) and fifty-two were female (36.5%). All of the isolates showed positive results for both biochemical and molecular identification tests.

**Table 2:** Demographic characteristics of patients enrolled in this study

Parameter	Clinical indications (*n* = 82)			
	NUD	PUD	IM	GC
No. of samples	52 (63.4%)	18 (21.9%)	11 (13.4%)	1 (1.2%)
Age range	40–80	40–60	20–60	60–70
Mean age ± SD	50.9 ± 4.39	44.4 ± 2.09	40.7 ± 3.51	63

NUD non-ulcer dyspepsia, PUD peptic ulcer disease, IM intestinal metaplasia, GC gastric cancer

### Screening for the clarithromycin sensitivity

The antibiotic susceptibility was assessed by examining the MIC values for all 82 isolates. The MIC assay demonstrated 42 (51.2%) clarithromycin-susceptible *H. pylori* isolates at the range of 0.06–0.125 µg/mL. The growth of 36 (43.9%) *H. pylori* isolates was unaffected by clarithromycin indicating resistant isolates with MICs ranging from 1 to 16 µg/mL. Few clinical specimens (4.9%), however, were intermediate to clarithromycin.

### Sequence analysis of 23S rRNA gene

From 82 clinical isolates, 48 isolates were selected for Sanger sequencing. We detected clarithromycin resistance-related point mutations in 27% (13/48) of *H. pylori* isolates. Showing the most important mutations related to clarithromycin resistance in Sanger sequencing, 11 *H. pylori* isolates were taken to pyrosequencing for comparing the results. The most frequent point mutation was the A2143G mutation detected in 4 out of 48 (8.3%) isolates. The prevalence of point mutations was followed by the A2142G mutation (6.2%), C2195T (4.1%), T2182C (4.1%), and C2288T (2%). The Sanger sequencing results of clarithromycin-resistant isolates are presented in Table 3.

**Table 3:** The results of Sanger sequencing for clarithromycin-resistant *H. pylori* isolates

Isolates	Clarithromycin susceptibility	MIC (mg/L)	Point mutations
HC70	Resistant	16	A2142G
HC133	Resistant	8	None
HC136	Resistant	8	A2142G
HC138	Resistant	16	None
HC160	Resistant	16	None
HC206	Resistant	8	None
OC4	Resistant	16	A2142G, G2220T, T2221C
OC15	Resistant	16	None
OC81	Resistant	1	A2624C
OC98	Resistant	8	C2248T
OC180	Resistant	16	A2143G
OC220	Resistant	8	None
OC245	Resistant	16	A2143G
OC254	Resistant	1	None
OC256	Resistant	1	None
OC258	Resistant	16	A2143G
OC359	Resistant	16	None
OC485	Resistant	2	None
OC557	Resistant	2	C2195T
OC797	Resistant	16	None
OC803	Resistant	1	T2182C
OC810	Resistant	2	C2195T
OC840	Resistant	1	A2143G
OC852	Resistant	2	C2288T
OC939	Resistant	4	None

### Comparison of the point mutations detection by the two methods

**Fig. 1:**
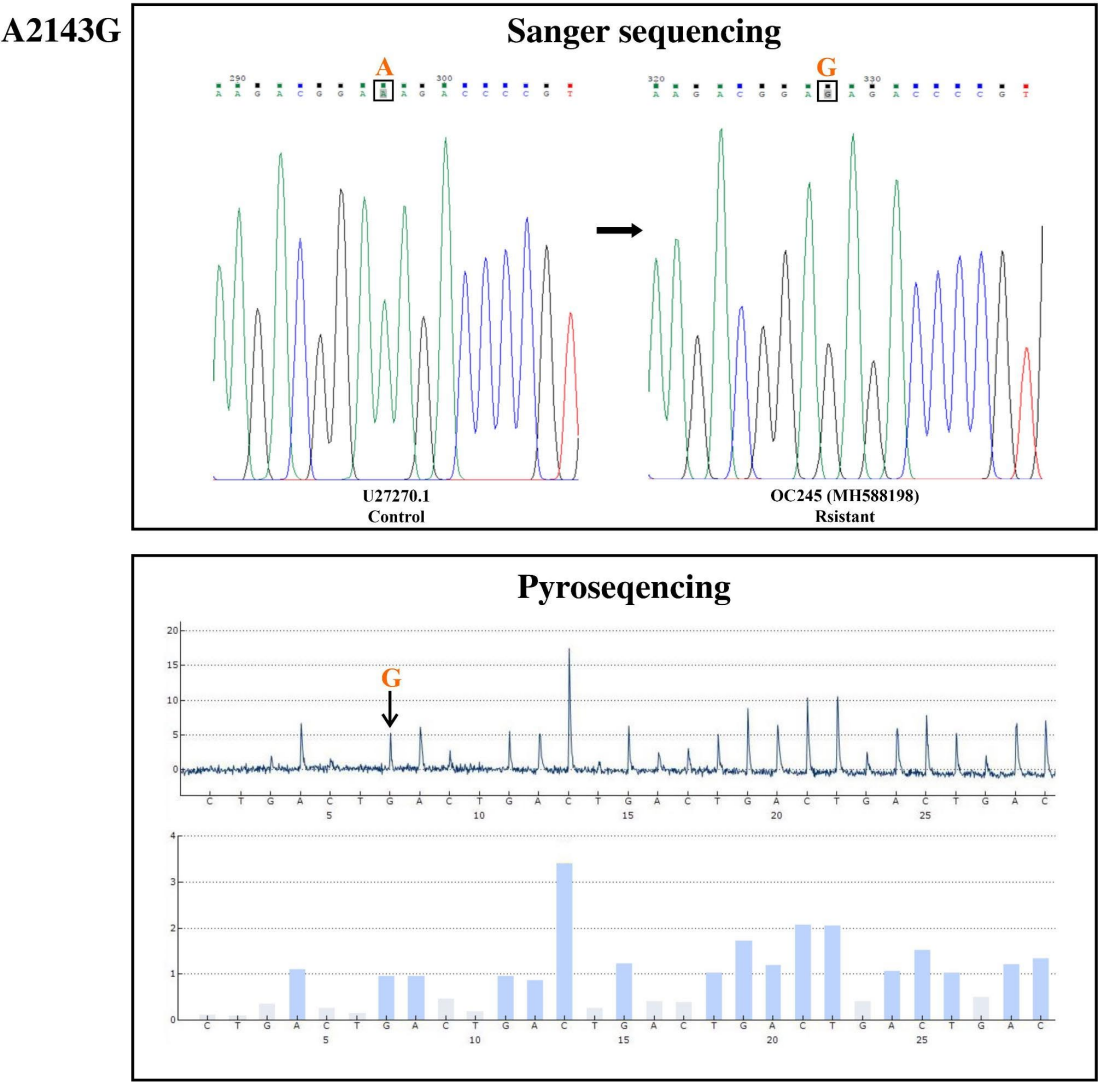
Sanger sequencing and pyrosequencing for A2143G mutation in a resistant *H. pylori* clinical isolate (OC245; accession number MH588198), compared with the U27270.1 control

**Fig. 2:**
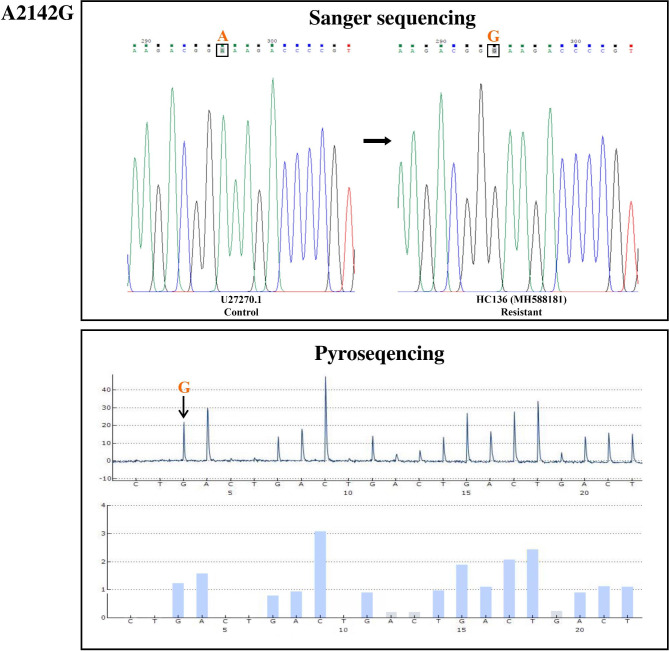
Sanger sequencing and pyrosequencing for A2142G mutation in a resistant *H. pylori* clinical isolate (HC136; accession number MH588181), compared with the U27270.1 control

Out of the 11 *H. pylori* isolates, 8 isolates were shown the same mutations in both sequencing methods, whereas 3 isolates demonstrated inconsistencies between pyrosequencing and Sanger sequencing platforms. A2142G and A2143G mutations were identified in both sequencing panels and the results had overlap with each other (Fig. 1 and 2). The C2195T mutation was only detected by the Sanger sequencing. The overall results and a comparison of the results from both platforms are summarized in Table 4.

**Table 4:** The results of genotypic detection for mutations associated with clarithromycin resistance in *H. pylori* isolates by the two methods

Isolates	Clarithromycin susceptibility	Mutations by Sanger sequencing	Mutations by pyrosequencing
HC70	Resistant	A2142G	A2142G
HC136	Resistant	A2142G	A2142G
OC4	Resistant	A2142G	A2142G
OC180	Resistant	A2143G	A2143G
OC245	Resistant	A2143G	A2143G
OC258	Resistant	A2143G	A2143G
OC557	Resistant	C2195T	ND
OC803	Resistant	T2182C	T2182C
OC810	Resistant	C2195T	ND
OC840	Resistant	A2143G	A2143G
OC852	Resistant	C2288T	C2288T

ND, not detected

## Discussion

*H. pylori* eradication has proven extremely challenging, as no monotherapy has the capacity to achieve sufficient (above 80%) efficacy. In clinical practice, only the combination of a proton pump inhibitor (PPI) and/or a bismuth component with two or three antibiotics (e.g., amoxicillin, clarithromycin, metronidazole, tetracycline, and levofloxacin) might effectively manage *H. pylori* infection [16, 17]. In addition to narrow treatment strategies, considerable uptake of particular antibiotics (such as clarithromycin) by the general public along with the rapid development of resistance mechanisms resulted in the emergence of primary antibiotic resistance in *H. pylori* [18, 19]. The increasing resistance of *H. pylori* to conventional therapeutic regimens has been reported worldwide for the past few decades, and Iran is among those countries in which resistance to the antibiotics of choice is alarmingly spreading across the country [14, 20, 21].

Clarithromycin is the most prescribed antibiotic in *H. pylori* treatment regimens, yet the increased rate of clarithromycin resistance has become a major issue in the efficacy of *H. pylori* eradication [4]. In the current study, the presence of clarithromycin-resistant *H. pylori* isolates was documented in Iran, and a pyrosequencing method for rapid detection of the related mutations was developed besides Sanger sequencing. In accordance with previous publications, we found 43.9% (36 out of 82 isolates) prevalence in *H. pylori* resistance to clarithromycin [22–24]. Therefore, based on the high prevalence of clarithromycin-resistant isolates, susceptibility testing is a reasonable manner prior to the use of clarithromycin, improving the eradication rate in *H. pylori-*infected patients.

Mechanistically, clarithromycin binds to the large subunit (50S) of ribosomes, targeting the 23S rRNA at the peptidyl transferase region. This action results in the inhibition of peptide transferase and the prevention of the peptide chain elongation, inhibiting bacterial protein synthesis [25]. The overwhelming majority of *H. pylori* clarithromycin-resistant strains present point mutations in the domain V of 23S rRNA (mostly A2143G, A2142G, and A2142C) [26, 27]. Prevention of clarithromycin attachment preserves the mRNA-tRNA translocation step for protein synthesis [28]. Furthermore, multidrug efflux pump systems constitute other potential mechanisms associated with the development of clarithromycin resistance [29, 30].

Our study confirmed the most frequent clarithromycin resistance-related mutations in *H. pylori* and provided reliable data by both Sanger and pyrosequencing platforms. Intriguingly both sequencing methods showed A2142G and A2143G as the most recurrent mutations among Iranian strains. However, pyrosequencing occasionally require confirmation by another platforms, as the C2195T mutation was only detected by Sanger sequencing that interrogates the entire sequence [31].

The focal point of the pyrosequencing assay in this study is to detect the most prevalent mutations related to clarithromycin resistance within a very short nucleotide sequence. There are several standard methods, including E-test, disk diffusion, agar dilution, and micro broth dilution, which are used to evaluate *H. pylori* resistance in clinical specimens. In comparison with the phenotypic methods, genotypic strategies such as sequencing, real-time PCR, and PCR-RFLP are less time-consuming and can identify resistance-inducing mutations [27].

## Conclusion

The current study demonstrates pyrosequencing as a rapid and effective method for the detection of clarithromycin-related mutations in *H. pylori* clinical isolates. Our results indicated a comparable accuracy between pyrosequencing and Sanger sequencing methods. The significant advantage of pyrosequencing is that it can be easily applied in clinical laboratories as a rapid and practical molecular diagnostic tool for screening and predicting the susceptibility profile of *H. pylori* isolates [32]. However, limitations in our study include the small number of sequenced isolates and the neglect of other point mutations associated with clarithromycin resistance and further antibiotics administered for *H. pylori* eradication namely fluoroquinolones. Therefore, further studies are required to investigate the potential for pyrosequencing in predicting the whole antibiotic resistance profile of *H. pylori*.

## Data Availability

All data generated or analyzed during this study are included in this published article.

## Abbreviations

bp: base pair
CLSI: Clinical and Laboratory Standards Institute
FCS: fetal calf serum
MIC: minimal inhibitory concentration
PPI: proton pump inhibitor
